# Directional Stimulus-Evoked Pallidal Electrophysiology in Primary Dystonia

**DOI:** 10.5334/tohm.916

**Published:** 2024-09-18

**Authors:** Aditya V. Boddu, Sarah Brinkerhoff, Adam E. Bashir, Camerron M. Crowder, Mohammed Awad, Christopher L. Gonzalez, Harrison C. Walker

**Affiliations:** 1 Department of Neurology, University of Arkansas for Medical Sciences, Little Rock, AR, USA; 2 Department of Neurology, University of Washington, Seattle, WA, USA; 3 Department of Neurobiology, University of Alabama at Birmingham, Birmingham, AL, USA; 4 Department of Electrical Engineering, University of Alabama at Birmingham, Birmingham, AL, USA; 5 Department of Neurology, University of Alabama at Birmingham, Birmingham, AL, USA; 6 Department of Biomedical Engineering, University of Alabama at Birmingham, Birmingham, AL, USA; 7 Department of Neurosurgery, University of Alabama at Birmingham, Birmingham, AL, USA

**Keywords:** dystonia, ERNA, deep brain stimulation, DBS, globus pallidus, ANO3

## Abstract

**Background:**

Deep brain stimulation for dystonia improves motor symptoms but variable and delayed responses challenge patient selection, targeting, and device programming.

**Case Report:**

Here we studied intracranial electrophysiology in a patient with primary dystonia and observed evoked resonant neural activity (ERNA) in the globus pallidus interna. These local stimulus-evoked potentials displayed refractory periods and paired-pulse facilitation at clinically relevant interstimulus intervals. Sensing from directional DBS contacts localized ERNA to an effective stimulation site in the ventral posterolateral portion of the pallidum.

**Discussion:**

To the best of our knowledge, this is the first observation of ERNA in the globus pallidus interna in a patient with primary dystonia. Stimulus-evoked activity could eventually guide both directional and adaptive stimulation for dystonia and other complex neuropsychiatric disorders.

## Introduction

Dystonia is characterized by abnormal postures and involuntary movements and can be associated with genetic etiologies. While deep brain stimulation (DBS) is a valuable therapy for various forms of primary dystonia, symptomatic responses can vary and require longer time intervals compared to other movement disorders indications [1,2,3,4]. This complicates patient selection, targeting, and programming with increasingly flexible and complex device technologies.

Biomarkers are needed to predict how deep brain stimulation (DBS) impacts target symptoms in patients with dystonia and other complex neurological disorders. With proper validation, these signals could inform contact selection or serve as control signals for adaptive stimulation with next-generation devices. One candidate biomarker is evoked resonant neural activity (ERNA) [5,6,7,8], a high-frequency, tapering sinusoidal local field potential elicited by the DBS pulse. ERNA is present in the subthalamic nucleus (STN) in patients with Parkinson’s disease (PD) [7,8], and its amplitude correlates with clinical efficacy. More recent studies show that STN stimulation elicits ERNA in patients with primary dystonia, as well [9]. We and others identified ERNA in the globus pallidus interna (GPi) in patients with PD [7,10], but whether it is present in patients with primary dystonia is unclear.

Here we report a patient with late, adult-onset, generalized dystonia with superimposed tremor who responded favourably to unilateral right pallidal DBS. Notably, contact selection by a blinded clinician corresponded spatially with evoked responses elicited by directional stimulation in the posterolateral pallidum.

## Case description

An 82-year-old right-handed woman of German ancestry first noted symptoms at age 40 with fist clenching and intermittent action tremors in the left hand. Over the next decade, she developed left arm flexion postures, involuntary eye blinking, and left face contractions. More recently she developed an occasional limp and stiffening with dragging of her feet. Associated abnormal postures were occasionally painful and worsened with voluntary movement. Medication trials with benzodiazepines, anticholinergics, opiates, muscle relaxants, levodopa, and carbamazepine yielded dose-limiting side effects without clear benefits. Neurological exam showed signs of generalized dystonia with left body predominance (see Supplementary Video). MR brain was unremarkable. Sequence analysis and deletion/duplication testing from the Invitae Dystonia Panel evaluating 18 genes disclosed a heterozygous variant of unknown significance in the anoctamin-3 gene (*ANO3*) at exon 27 c.2812G>A (p.Val938Ile).

**Supplementary Video:**
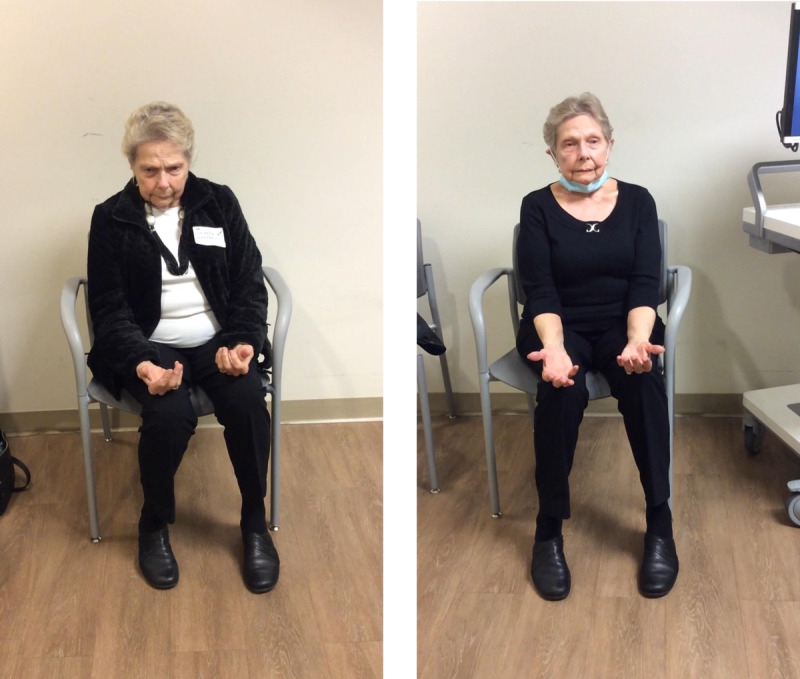
Videos compare movements before versus after DBS (left versus right panels) following unilateral right GPi DBS. Pre-treatment video shows fine, irregular postural and intention tremor on the left more than the right body, associated with abnormal postures. Involuntary eye-blinking (blepharospasm) occurs predominantly during voluntary movements, and left-hand postures persist during gait. Spiral drawing with the left hand is challenged by irregular, jerky tremor. Post-treatment video demonstrates improvements in blepharospasm, tremor, left-hand posture, and writing (https://doi.org/10.5334/tohm.916.s1).

## Methods

**Figure 1:**
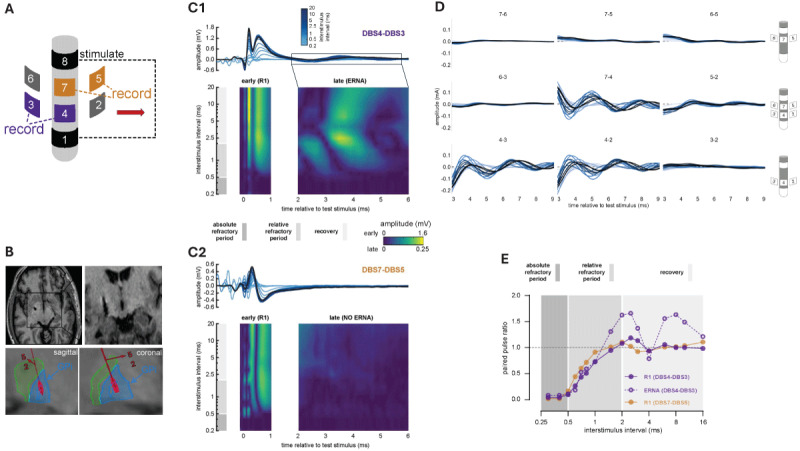
**Stimulus-evoked potentials recorded from directional DBS contacts in the globus pallidus interna in a patient with primary dystonia. (A)** *S*chema of stimulation and recording sites on the DBS lead. Red arrow refers to the spatial orientation of the arrows in panel B. **(B)** Axial and anterior T1-weighted brain MR images and Brainlab reconstructions showing directional lead orientation in the pallidum. Red arrows point in the orientation of DBS contacts 2 and 5. **(C1–C2)** Raster and contour plots after artifact removal show that the early and later components of the DBS-evoked GPi potentials both display relative and absolute refractory periods at paired pulse intervals of 0.5 to 2 ms and <0.5 ms, respectively. R1 is larger in amplitude and detected at both recording sites, whereas ERNA is not detected when recording from DBS contact segments 5 and 7. **(D)** Raster plots across different paired pulse intervals show maximal detection of ERNA from directional DBS contacts in the ventral lateral portion of the pallidum. Response amplitudes and phase reversal suggest that directional contact 4 is most proximal to the source of the signal. **(E)** R1 and ERNA both show absolute and relative refractory periods, but ERNA alone displays short-term plasticity with paired pulse ratios significantly greater than 1 at multiple interstimulus intervals (>1.5 ms).

Because of her relatively advanced age and left-sided motor predominance, we recommended unilateral right globus pallidum interna (GPi) DBS (Figure 1A). Routine clinical practice guided lead placement, which combined information from multi-pass microelectrode recordings, macrostimulation, intraoperative O-arm 2 imaging, and real-time reconstructions in BrainLAB.

With prior informed consent and IRB approval, stimulation and recordings were performed intraoperatively after brain mapping with the DBS electrode in its final location with the patient awake and at rest. All experimental stimuli were physically imperceptible. Stimuli were delivered through a ‘1–3-3-1’ Boston Scientific DBS lead via an externalized, battery-powered pulse generator disconnected from the wall outlet. Bipolar stimuli were delivered from contacts 4 and 1 on the implanted DBS electrode. Monophasic stimuli were employed because they most closely resemble clinical stimulus pulses. We achieved charge balance by alternating cathode and anode phase stimuli; either on a pulse-by-pulse basis for single pulses or after two consecutive stimuli for pairs of pulses. Thus, no significant charge accumulation could occur. The pulse generator sent a TTL (transistor-transistor logic) pulse at the onset of each stimulus to the digital-in channel on the recording amplifier, allowing immediate reconstruction of event related potentials. We sampled at 100 kHz, yielding sync precision of 10 µsec.

We evaluated responses to single and paired stimuli. Pairs of stimuli have been employed in neurophysiology studies for decades because they can provide information on neural refractoriness and short-term plasticity [14]. Here we utilized paired stimuli to examine the fast dynamics of local stimulus-evoked physiology in the context of DBS therapy. Block randomization ensured that all interstimulus intervals were delivered in random order on a block-by-block basis. The first pulse (conditioning stimulus) is followed by a second pulse (test stimulus). We utilized 16 paired pulse intervals ranging from 0.2 to 16 milliseconds. These intervals correspond to anticipated neural refractory periods (<2 milliseconds) and timing relevant to clinical DBS frequencies (~5–10 milliseconds). Stimulus pairs were separated by pauses of 45 to 55 milliseconds. The stimulus amplitude and pulse width were 2.6 mA and 60 µsec, respectively, which corresponds to parameters used for trial macrostimulation with the implanted lead (i.e., 4 V, 60 µsec, 160 Hz).

Stimulus-evoked potentials were analysed after averaging time-locked epochs surrounding each stimulation event. Stimulus artifact and residual signals from prior conditioning stimuli were removed by template subtraction, as described previously [7]. This yielded sets of DBS-evoked potentials recorded in parallel from each of the 6 directional contacts. The residual potentials were visualized in raster form with traces and contour plots (Figure 1B), and paired pulse ratio was calculated by dividing the test stimulus response by the conditioning stimulus response (Figure 1C). A paired pulse ratio equal to 0 indicates that the test stimulus elicited no response, whereas a ratio of 1 indicates that the test and conditioning stimuli elicited identical responses. Paired pulse ratio >1 or <1 indicate stronger or weaker responses to the conditioning stimulus.

## Results

Figure 1A provides an overall schema and anatomical rendering of the orientation of the directional DBS electrode array in the pallidum. Figure 1B demonstrates local stimulus-evoked potentials after artifact removal in the globus pallidus interna. The earliest response (termed R1) is large in amplitude, begins <0.5 ms after stimulus onset, and is almost entirely obscured by the stimulus transient prior to artifact removal [7]. Later responses (ERNA) begin at >2 ms latency and consist of multiple oscillatory peaks over a more prolonged time interval. Importantly, R1 and ERNA both display relative and absolute refractory periods at the shortest interstimulus intervals (<2 ms). DBS contacts 3–4 that were oriented spatially towards the ventral posterolateral pallidum captured ERNA, whereas recordings from contacts 5–7 did not. Notably, ERNA but not R1 displayed paired pulse facilitation with paired pulse ratios >1 at multiple interstimulus intervals (Figure 1C). The orientation of the lead was measured using the FDA-approved BrainLAB software suite.

We examined the behavioural effects of stimulation from all contacts on the DBS electrode during initial device activation in clinic. The clinician who programmed the device was unaware of the spatial distribution of the DBS-evoked electrophysiology at the time of initial device programming. The best efficacy and side effect thresholds during this session arose from DBS row 2 (i.e., the most ventral directional row). Based upon its acute efficacy and reasonable location on postoperative imaging, we selected the contacts on this row for chronic therapy. The patient experienced mild but definite improvements in involuntary hand postures, eye blinking during voluntary movements, tremor, and gait over the subsequent weeks to months. Video ratings of the Burke Fahn Marsden dystonia rating scale improved from a preoperative baseline of 14 to 10 at nine months after unilateral DBS (see Supplementary Video). The most meaningful functional gains related to activities requiring hand dexterity. Similar improvements were noted at 4 months after surgery with identical stimulator settings, as well. Conventional programming settings were employed with monopolar stimulation from the ventral row of segmented contacts with 4 mA amplitude, 60 µsec pulse width, and 143 Hz frequency. Thus, amplitude and pulse width of her therapeutic stimulation were similar to those employed during the intraoperative experiments. At her most recent clinical encounter 1 year after surgery, pulse width and amplitude were further increased, however no additional follow-up was possible, as she expired from complications of community acquired pneumonia at approximately 2 years after surgery.

## Discussion

Here we report a patient with primary generalized dystonia whose motor symptoms improved with globus pallidus interna brain stimulation. Directional DBS contacts detected evoked recurrent neural activity (ERNA) in the posterolateral pallidum at a site that was later selected for therapy by a clinician blinded to the intraoperative electrophysiology. As best as we are aware, this is the first observation of ERNA in the GPi in a patient with primary dystonia, building on prior results from the pallidum in the context of PD [7,10].

Local stimulus-evoked activity shows promise as a candidate biomarker to guide both electrode targeting and open- and closed-loop approaches to DBS [11]. Here we provide additional evidence that directional sensing can identify the spatial orientation of ERNA around the stimulation site (Figure 1A–B). Another group found that ERNA amplitude in the subthalamic nucleus correlates with the motor efficacy of DBS for PD [12]. Similarly, pallidal ERNA appears useful for guiding targeting and stimulator programming [10], which complements findings in this case that the spatial distribution of ERNA during surgery corresponds with contacts later selected for chronic therapy.

Causal mechanisms underlying these local stimulus-evoked potentials remain unclear. Subthalamic and pallidal stimulation yield ERNA in patients with both PD [7,8,10] and primary dystonia [9], whereas ventral intermediate thalamus stimulation does not [7,8]. ERNA, therefore, appears to be a feature of stimulus-evoked activity in the subthalamic-pallidal circuit that does not relate specifically to either disease state. Although ventral intermediate nucleus (VIM) stimulation reduces tremor, ERNA is not detected when stimulating and recording from the VIM in patients with essential tremor [13]. Regarding underlying mechanisms, R1 and ERNA both display absolute and relative refractory periods, which validates their neural origin. The short latency and large amplitude of R1 implies direct non-synaptic activation of local neural elements [7], whereas the more delayed onset and short-term plasticity in the ERNA response both suggest synaptic mechanisms (Figure 1C).

Short-term plasticity refers to immediate changes in neural responses that could reflect either increases (paired pulse facilitation) or decreases (paired pulse depression) in a stimulus-evoked potential. Here, and in a prior study in the STN [7], we found that DBS elicits paired pulse facilitation at specific interstimulus intervals. The largest paired pulse facilitation in this report and in our prior study [7] occurred at interstimulus intervals of ~2 ms, which would correspond to continuous frequency of 500 Hz. Although this relatively high stimulation frequency is unavailable on commercial DBS devices, we also found facilitation at wider interstimulus intervals (5–10 ms) that correspond to clinically effective stimulation frequencies (i.e., 100–200 Hz). Interestingly, interstimulus intervals of >10 ms elicit less facilitation and correspond with clinically ineffective stimulation frequencies. Whether and how paired pulse facilitation in GPi and STN evolves over longer trains of stimuli should be investigated in future studies. If paired pulse facilitation were found to predict clinical efficacy in a larger sample, a future DBS device might use pairs or bursts of stimuli to generate tuning curves to identify suitable locations and/or frequencies for therapeutic stimulation within individuals.

## Limitations

Current implanted DBS hardware with sensing capabilities can record beta activity (13–30 Hz) and other low frequency components of spontaneous or task-based local field potentials. However, these devices do not sample rapidly enough to accurately capture the fastest components of ERNA and other stimulus-evoked potential waveforms. Next-generation DBS devices with greater bandwidth are needed, as the stimulus-evoked physiology could potentially inform new approaches to targeting during surgery and both open and closed-loop neuromodulation in the ambulatory setting. Conclusions about the prevalence of ERNA in the pallidum in patients with primary dystonia and whether DBS-evoked electrophysiology is linked to clinical efficacy should be drawn with some caution as this is a case report. Prospective studies are needed to examine whether ERNA amplitude correlates with relief of motor symptoms in patients with dystonia and other movement disorders.

## Conclusion

This case expands knowledge on directional stimulus-evoked activity in the globus pallidus interna in a patient with primary dystonia. Future studies should prospectively examine whether local DBS electrophysiology could serve as a control signal to guide therapy for patients with disabling motor symptoms from dystonia and other complex neurological disorders.

## Additional File

The additional file for this article can be found as follows:

10.5334/tohm.916.s1 Supplementary Video. Videos compare movements before versus after DBS (left versus right panels) following unilateral right GPi DBS.
