# Supplementary figures and images for: Directional Stimulus-Evoked Pallidal Electrophysiology in Primary Dystonia
Source: Tremor Other Hyperkinet Mov (N Y). 2024 Sep 18;14:46. doi: 10.5334/tohm.916 (PMC11414461; doi:10.5334/tohm.916)

## Slide 1
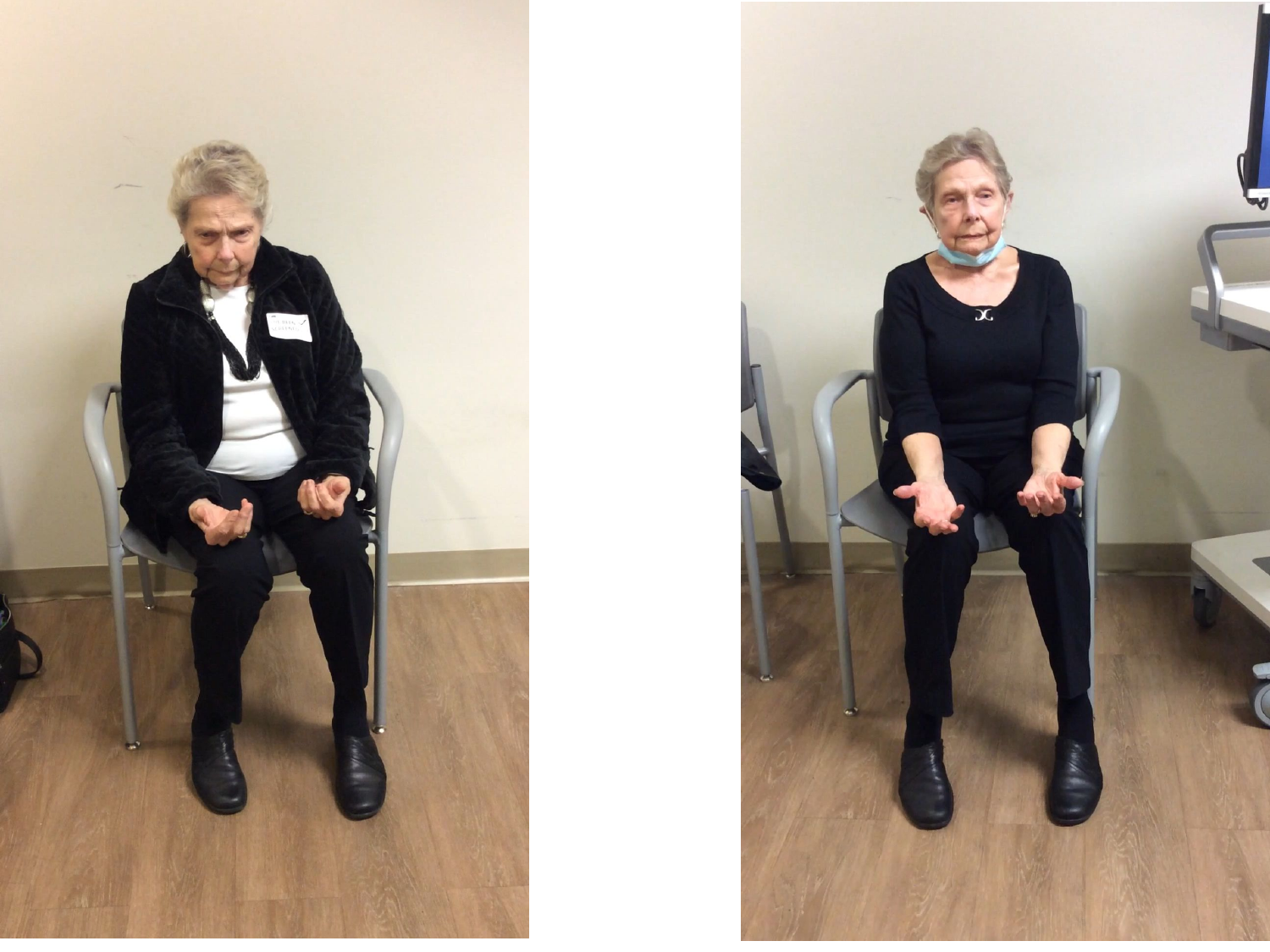

Supplement: Supplementary Video. — Videos compare movements before versus after DBS (left versus right panels) following unilateral right GPi DBS. [file tohm-14-1-916-s1.pptx]
